# Supplementary material for: Skeletal Muscle Gene Expression Profile in Response to Caloric Restriction and Aging: A Role for SirT1
Source: Genes (Basel). 2021 May 5;12(5):691. doi: 10.3390/genes12050691 (PMC8147962; doi:10.3390/genes12050691)
Supplement: Supplementary file 1 [file genes-12-00691-s001.zip › Figure S1.pdf.zip!/Figure S1.pdf]

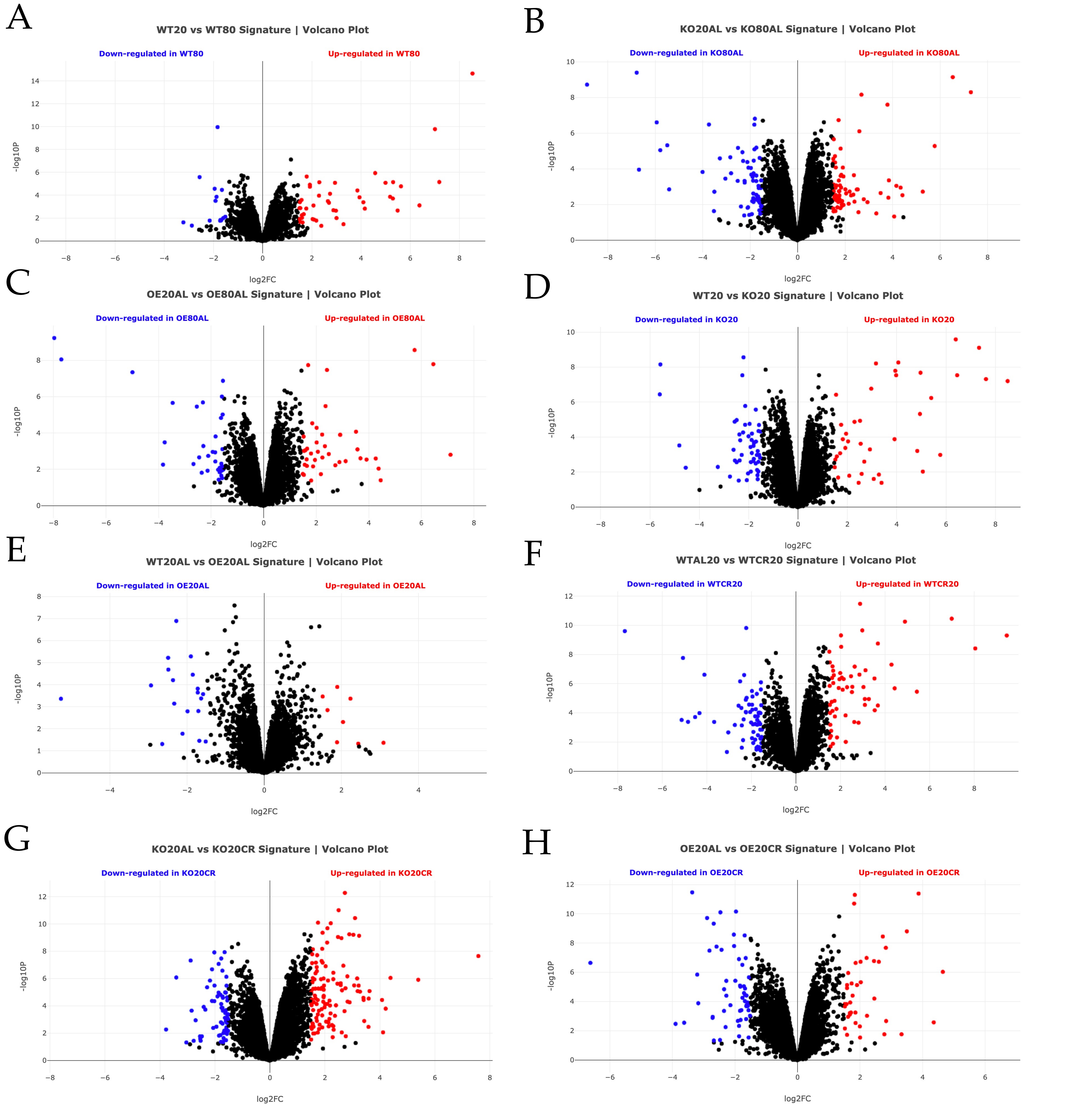
A
WT20 vs WT80 Signature | Volcano Plot
Down-regulated in WT80
Up-regulated in WT80
B
KO20AL vs KO80AL Signature | Volcano Plot
Down-regulated in KO80AL
Up-regulated in KO80AL
C
OE20AL vs OE80AL Signature | Volcano Plot
Down-regulated in OE80AL
Up-regulated in OE80AL
D
WT20 vs KO20 Signature | Volcano Plot
Down-regulated in KO20
Up-regulated in KO20
E
WT20AL vs OE20AL Signature | Volcano Plot
Down-regulated in OE20AL
Up-regulated in OE20AL
F
WTAL20 vs WTCR20 Signature | Volcano Plot
Down-regulated in WTCR20
Up-regulated in WTCR20
G
KO20AL vs KO20CR Signature | Volcano Plot
Down-regulated in KO20CR
Up-regulated in KO20CR
H
OE20AL vs OE20CR Signature | Volcano Plot
Down-regulated in OE20CR
Up-regulated in OE20CR
-log10P
log2FC

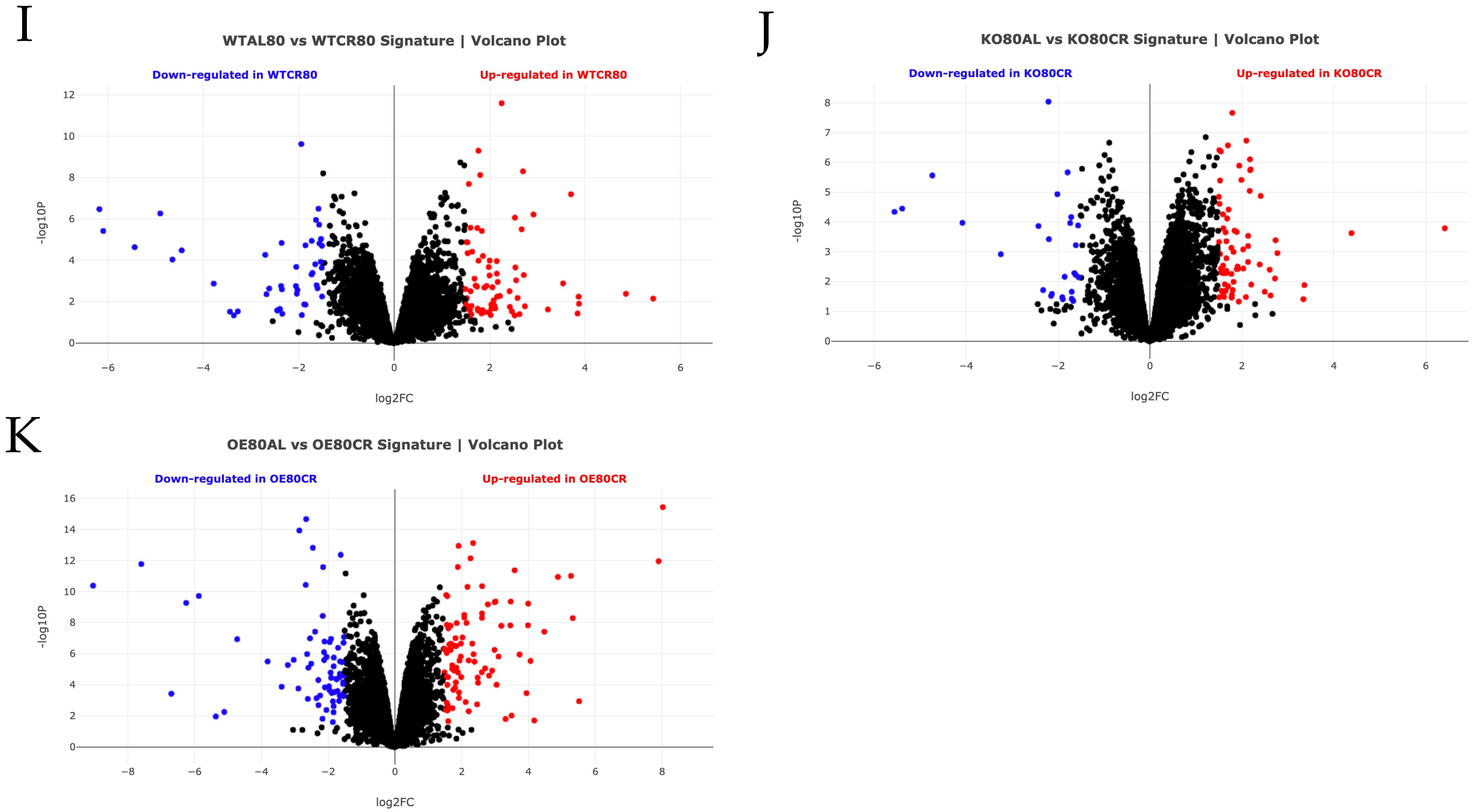
I
WTAL80 vs WTCR80 Signature | Volcano Plot
Down-regulated in WTCR80
Up-regulated in WTCR80
-log10P
log2FC
J
KO80AL vs KO80CR Signature | Volcano Plot
Down-regulated in KO80CR
Up-regulated in KO80CR
-log10P
log2FC
K
OE80AL vs OE80CR Signature | Volcano Plot
Down-regulated in OE80CR
Up-regulated in OE80CR
-log10P
log2FC
