# Supplementary material for: Skeletal Muscle Gene Expression Profile in Response to Caloric Restriction and Aging: A Role for SirT1
Source: Genes (Basel). 2021 May 5;12(5):691. doi: 10.3390/genes12050691 (PMC8147962; doi:10.3390/genes12050691)
Supplement: Supplementary file 1 [file genes-12-00691-s001.zip › Suppl_tables_S1-S13.zip!/Table S3-S13.docx]

**Table S3.** Top 50 significantly regulated biological pathways in response to aging in WT mice.

| **No** | **Pathway** | **GO term** | **log10P** | **Regulation** |
| --- | --- | --- | --- | --- |
| *WT80* | | | | |
| 1 | muscle filament sliding (GO:0030049) | BP | 7.66 | up |
| 2 | actin-myosin filament sliding (GO:0033275) | BP | 7.66 | up |
| 3 | collagen fibril organization (GO:0030199) | BP | 7.64 | down |
| 4 | muscle contraction (GO:0006936) | BP | 7.23 | up |
| 5 | platelet-derived growth factor binding (GO:0048407) | MF | 7.01 | down |
| 6 | protein complex subunit organization (GO:0071822) | BP | 5.85 | down |
| 7 | SRP-dependent co-translational protein targeting to membrane (GO:0006614) | BP | 5.66 | up |
| 8 | striated muscle contraction (GO:0006941) | BP | 5.60 | up |
| 9 | skeletal muscle contraction (GO:0003009) | BP | 5.47 | up |
| 10 | Co-translational protein targeting to membrane (GO:0006613) | BP | 5.46 | up |
| 11 | nuclear-transcribed mRNA catabolic process, nonsense-mediated decay (GO:0000184) | BP | 5.34 | up |
| 12 | protein targeting to ER (GO:0045047) | BP | 5.27 | up |
| 13 | cellular response to DNA damage stimulus (GO:0006974) | BP | 5.00 | down |
| 14 | heart contraction (GO:0060047) | BP | 4.94 | up |
| 15 | viral gene expression (GO:0019080) | BP | 4.70 | up |
| 16 | actin binding (GO:0003779) | MF | 4.63 | up |
| 17 | viral transcription (GO:0019083) | BP | 4.58 | up |
| 18 | regulation of energy homeostasis (GO:2000505) | BP | 4.48 | down |
| 19 | negative regulation of cell proliferation (GO:0008285) | BP | 4.32 | up |
| 20 | ubiquitin-protein transferase activity (GO:0004842) | MF | 4.26 | down |
| 21 | microfilament motor activity (GO:0000146) | MF | 4.20 | up |
| 22 | cytosolic ribosome (GO:0022626) | CC | 4.18 | up |
| 23 | ribosome (GO:0005840) | CC | 3.94 | up |
| 24 | nuclear-transcribed mRNA catabolic process (GO:0000956) | BP | 3.90 | up |
| 25 | cellular glucose homeostasis (GO:0001678) | BP | 3.83 | down |
| 26 | myofibril assembly (GO:0030239) | BP | 3.81 | up |
| 27 | protein ubiquitination (GO:0016567) | BP | 3.70 | down |
| 28 | regulation of I-kappaB kinase/NF-kappaB signaling (GO:0043122) | BP | 3.70 | up |
| 29 | positive regulation of ATP metabolic process (GO:1903580) | BP | 3.65 | down |
| 30 | cardiac muscle tissue morphogenesis (GO:0055008) | BP | 3.61 | up |
| 31 | cardiac muscle contraction (GO:0060048) | BP | 3.61 | up |
| 32 | muscle cell differentiation (GO:0042692) | BP | 3.61 | up |
| 33 | cytoskeleton (GO:0005856) | CC | 3.52 | up |
| 34 | Cul3-RING ubiquitin ligase complex (GO:0031463) | CC | 3.46 | down |
| 35 | actin filament binding (GO:0051015) | MF | 3.46 | up |
| 36 | regulation of muscle contraction (GO:0006937) | BP | 3.39 | up |
| 37 | vasculature development (GO:0001944) | BP | 3.37 | down |
| 38 | actin cytoskeleton (GO:0015629) | CC | 3.33 | up |
| 39 | transcription regulatory region DNA binding (GO:0044212) | MF | 3.29 | up |
| 40 | rRNA metabolic process (GO:0016072) | BP | 3.28 | up |
| 41 | polysomal ribosome (GO:0042788) | CC | 3.23 | up |
| 42 | rRNA processing (GO:0006364) | BP | 3.23 | up |
| 43 | transcription corepressor activity (GO:0003714) | MF | 3.21 | up |
| 44 | cytosolic part (GO:0044445) | CC | 3.17 | up |
| 45 | extracellular matrix organization (GO:0030198) | BP | 3.17 | down |
| 46 | sarcoplasmic reticulum (GO:0016529) | CC | 3.16 | up |
| 47 | striated muscle tissue development (GO:0014706) | BP | 3.16 | up |
| 48 | WW domain binding (GO:0050699) | MF | 3.16 | up |
| 49 | positive regulation of cartilage development (GO:0061036) | BP | 3.15 | down |
| 50 | skeletal muscle thin filament assembly (GO:0030240) | BP | 3.10 | up |

**Table S4.** Top 50 significantly regulated biological pathways in response to aging in SirT1 KO mice.

| **No** | **Pathway** | **GO term** | **log10P** | **Regulation** |
| --- | --- | --- | --- | --- |
| *KO80* | | | | |
| 1 | regulation of transcription from RNA polymerase II promoter (GO:0006357) | BP | 6.78 | up |
| 2 | transcription regulatory region sequence-specific DNA binding (GO:0000976) | MF | 6.43 | up |
| 3 | nuclear-transcribed mRNA catabolic process (GO:0000956) | BP | 6.40 | up |
| 4 | viral gene expression (GO:0019080) | BP | 6.20 | up |
| 5 | RNA polymerase II regulatory region DNA binding (GO:0001012) | MF | 6.12 | up |
| 6 | regulation of fat cell differentiation (GO:0045598) | BP | 5.79 | up |
| 7 | positive regulation of transcription, DNA-templated (GO:0045893) | BP | 5.53 | up |
| 8 | viral process (GO:0016032) | BP | 5.53 | up |
| 9 | positive regulation of transcription from RNA polymerase II promoter (GO:0045944) | BP | 5.38 | up |
| 10 | transcription coactivator activity (GO:0003713) | MF | 5.35 | up |
| 11 | nuclear-transcribed mRNA catabolic process, nonsense-mediated decay (GO:0000184) | BP | 5.34 | up |
| 12 | viral transcription (GO:0019083) | BP | 5.30 | up |
| 13 | connective tissue development (GO:0061448) | BP | 5.14 | up |
| 14 | peptide biosynthetic process (GO:0043043) | BP | 5.09 | up |
| 15 | positive regulation of cell differentiation (GO:0045597) | BP | 5.09 | up |
| 16 | protein phosphorylation (GO:0006468) | BP | 5.08 | up |
| 17 | mitochondrial ATP synthesis coupled electron transport (GO:0042775) | BP | 5.07 | down |
| 18 | regulation of intracellular signal transduction (GO:1902531) | BP | 5.05 | up |
| 19 | AP-2 adaptor complex binding (GO:0035612) | MF | 4.90 | up |
| 20 | nucleoplasm part (GO:0044451) | CC | 4.87 | up |
| 21 | SRP-dependent cotranslational protein targeting to membrane (GO:0006614) | BP | 4.87 | up |
| 22 | cytosolic ribosome (GO:0022626) | CC | 4.85 | up |
| 23 | transcription corepressor activity (GO:0003714) | MF | 4.83 | up |
| 24 | peptidyl-serine phosphorylation (GO:0018105) | BP | 4.77 | up |
| 25 | cotranslational protein targeting to membrane (GO:0006613) | BP | 4.69 | up |
| 26 | histone methyltransferase activity (GO:0042054) | MF | 4.65 | up |
| 27 | basement membrane organization (GO:0071711) | BP | 4.60 | down |
| 28 | protein targeting to ER (GO:0045047) | BP | 4.51 | up |
| 29 | protein autophosphorylation (GO:0046777) | BP | 4.45 | up |
| 30 | positive regulation of fat cell differentiation (GO:0045600) | BP | 4.35 | up |
| 31 | cytosolic part (GO:0044445) | CC | 4.32 | up |
| 32 | mitochondrion (GO:0005739) | CC | 4.32 | down |
| 33 | ncRNA processing (GO:0034470) | BP | 4.21 | up |
| 34 | histone-lysine N-methyltransferase activity (GO:0018024) | MF | 4.20 | up |
| 35 | transcription regulatory region DNA binding (GO:0044212) | MF | 4.13 | up |
| 36 | negative regulation of transcription, DNA-templated (GO:0045892) | BP | 4.08 | up |
| 37 | nuclear body (GO:0016604) | CC | 4.04 | up |
| 38 | negative regulation of transcription from RNA polymerase II promoter (GO:0000122) | BP | 4.02 | up |
| 39 | peptidyl-serine modification (GO:0018209) | BP | 4.01 | up |
| 40 | RNA polymerase II regulatory region sequence-specific DNA binding (GO:0000977) | MF | 3.98 | up |
| 41 | cell junction assembly (GO:0034329) | BP | 3.98 | down |
| 42 | ribosome (GO:0005840) | CC | 3.94 | up |
| 43 | sequence-specific double-stranded DNA binding (GO:1990837) | MF | 3.94 | up |
| 44 | respiratory electron transport chain (GO:0022904) | BP | 3.92 | down |
| 45 | cellular response to hexose stimulus (GO:0071331) | BP | 3.85 | up |
| 46 | cellular glucose homeostasis (GO:0001678) | BP | 3.83 | up |
| 47 | mitochondrial inner membrane (GO:0005743) | CC | 3.83 | down |
| 48 | PML body (GO:0016605) | CC | 3.83 | up |
| 49 | protein-lysine N-methyltransferase activity (GO:0016279) | MF | 3.81 | up |
| 50 | core promoter binding (GO:0001047) | MF | 3.80 | up |

**Table S5.** Top 50 significantly regulated biological pathways in response to aging in SirT1 OE mice.

| **No** | **Pathway** | **GO term** | **log10P** | **Regulation** |
| --- | --- | --- | --- | --- |
| *OE80* | | | | |
| 1 | regulation of apoptotic process (GO:0042981) | BP | 6.22 | down |
| 2 | response to unfolded protein (GO:0006986) | BP | 5.01 | down |
| 3 | regulation of cell migration (GO:0030334) | BP | 4.78 | down |
| 4 | negative regulation of signal transduction (GO:0009968) | BP | 4.49 | down |
| 5 | regulation of angiogenesis (GO:0045765) | BP | 4.39 | down |
| 6 | transcription regulatory region sequence-specific DNA binding (GO:0000976) | MF | 4.30 | up |
| 7 | positive regulation of extrinsic apoptotic signaling pathway (GO:2001238) | BP | 4.25 | down |
| 8 | cytoplasmic vesicle lumen (GO:0060205) | CC | 4.01 | down |
| 9 | positive regulation of cellular metabolic process (GO:0031325) | BP | 4.00 | down |
| 10 | positive regulation of cellular process (GO:0048522) | BP | 3.90 | down |
| 11 | core promoter binding (GO:0001047) | MF | 3.80 | up |
| 12 | regulation of blood vessel size (GO:0050880) | BP | 3.79 | down |
| 13 | skeletal muscle cell differentiation (GO:0035914) | BP | 3.79 | down |
| 14 | voltage-gated cation channel activity (GO:0022843) | MF | 3.69 | up |
| 15 | glutathione metabolic process (GO:0006749) | BP | 3.69 | down |
| 16 | lipoprotein particle binding (GO:0071813) | MF | 3.65 | down |
| 17 | ficolin-1-rich granule (GO:0101002) | CC | 3.64 | down |
| 18 | negative regulation of apoptotic process (GO:0043066) | BP | 3.62 | down |
| 19 | positive regulation of angiogenesis (GO:0045766) | BP | 3.59 | down |
| 20 | voltage-gated potassium channel activity (GO:0005249) | MF | 3.57 | up |
| 21 | post-translational protein modification (GO:0043687) | BP | 3.56 | down |
| 22 | positive regulation of vasculature development (GO:1904018) | BP | 3.56 | down |
| 23 | positive regulation of apoptotic process (GO:0043065) | BP | 3.54 | down |
| 24 | positive regulation of extrinsic apoptotic signaling pathway via death domain receptors (GO:1902043) | BP | 3.53 | down |
| 25 | transforming growth factor beta-activated receptor activity (GO:0005024) | MF | 3.53 | down |
| 26 | cytokine-mediated signaling pathway (GO:0019221) | BP | 3.51 | down |
| 27 | regulation of extrinsic apoptotic signaling pathway via death domain receptors (GO:1902041) | BP | 3.42 | down |
| 28 | lipoprotein particle receptor activity (GO:0030228) | MF | 3.37 | down |
| 29 | negative regulation of insulin secretion (GO:0046676) | BP | 3.37 | up |
| 30 | Rab GTPase binding (GO:0017137) | MF | 3.37 | up |
| 31 | core promoter sequence-specific DNA binding (GO:0001046) | MF | 3.33 | up |
| 32 | transcription regulatory region DNA binding (GO:0044212) | MF | 3.29 | up |
| 33 | neutrophil activation involved in immune response (GO:0002283) | BP | 3.28 | down |
| 34 | negative regulation of peptide hormone secretion (GO:0090278) | BP | 3.26 | up |
| 35 | regulation of cell growth (GO:0001558) | BP | 3.25 | down |
| 36 | GTPase activator activity (GO:0005096) | MF | 3.25 | up |
| 37 | negative regulation of protein secretion (GO:0050709) | BP | 3.24 | up |
| 38 | positive regulation of cell motility (GO:2000147) | BP | 3.23 | down |
| 39 | neutrophil mediated immunity (GO:0002446) | BP | 3.23 | down |
| 40 | GTPase regulator activity (GO:0030695) | MF | 3.23 | up |
| 41 | positive regulation of apoptotic signaling pathway (GO:2001235) | BP | 3.21 | down |
| 42 | positive regulation of cellular biosynthetic process (GO:0031328) | BP | 3.17 | down |
| 43 | focal adhesion (GO:0005925) | CC | 3.16 | down |
| 44 | regulation of myoblast differentiation (GO:0045661) | BP | 3.16 | down |
| 45 | delayed rectifier potassium channel activity (GO:0005251) | MF | 3.16 | up |
| 46 | positive regulation of cellular protein catabolic process (GO:1903364) | BP | 3.15 | down |
| 47 | low-density lipoprotein particle binding (GO:0030169) | MF | 3.15 | down |
| 48 | regulation of smooth muscle cell proliferation (GO:0048660) | BP | 3.13 | down |
| 49 | positive regulation of transport (GO:0051050) | BP | 3.13 | down |
| 50 | cellular protein modification process (GO:0006464) | BP | 3.12 | down |

**Table S6.** Top 50 significantly regulated biological pathways in response to SirT1 KO in young mice.

| **No** | **Pathway** | **GO term** | **log10P** | **Regulation** |
| --- | --- | --- | --- | --- |
| *SirT1 knockout in young mice* | | | | |
| 1 | RNA binding (GO:0003723) | MF | 8.22 | up |
| 2 | muscle contraction (GO:0006936) | BP | 7.23 | down |
| 3 | mitochondrial ATP synthesis coupled electron transport (GO:0042775) | BP | 6.74 | up |
| 4 | mitochondrial respiratory chain complex I (GO:0005747) | CC | 6.35 | up |
| 5 | respiratory electron transport chain (GO:0022904) | BP | 6.22 | up |
| 6 | mRNA splicing, via spliceosome (GO:0000398) | BP | 6.13 | up |
| 7 | mRNA processing (GO:0006397) | BP | 6.10 | up |
| 8 | NADH dehydrogenase (quinone) activity (GO:0050136) | MF | 6.03 | up |
| 9 | NADH dehydrogenase (ubiquinone) activity (GO:0008137) | MF | 6.03 | up |
| 10 | substantia nigra development (GO:0021762) | BP | 6.03 | up |
| 11 | mitochondrial electron transport, NADH to ubiquinone (GO:0006120) | BP | 5.77 | up |
| 12 | muscle organ development (GO:0007517) | BP | 5.67 | down |
| 13 | RNA splicing, via transesterification reactions with bulged adenosine as nucleophile (GO:0000377) | BP | 5.66 | up |
| 14 | regulation of cellular amine metabolic process (GO:0033238) | BP | 5.37 | up |
| 15 | regulation of cellular amino acid metabolic process (GO:0006521) | BP | 5.37 | up |
| 16 | mitochondrial inner membrane (GO:0005743) | CC | 5.24 | up |
| 17 | focal adhesion (GO:0005925) | CC | 4.93 | down |
| 18 | elastic fiber assembly (GO:0048251) | BP | 4.90 | down |
| 19 | regulation of cellular ketone metabolic process (GO:0010565) | BP | 4.71 | up |
| 20 | muscle contraction (GO:0006936) | BP | 4.39 | up |
| 21 | insulin receptor signaling pathway (GO:0008286) | BP | 4.38 | down |
| 22 | positive regulation of fat cell differentiation (GO:0045600) | BP | 4.35 | down |
| 23 | mRNA binding (GO:0003729) | MF | 4.34 | up |
| 24 | positive regulation of protein ubiquitination involved in ubiquitin-dependent protein catabolic process (GO:2000060) | BP | 4.21 | up |
| 25 | DNA-templated transcription, termination (GO:0006353) | BP | 4.16 | up |
| 26 | cellular response to insulin stimulus (GO:0032869) | BP | 4.01 | down |
| 27 | sarcoplasm (GO:0016528) | CC | 3.99 | down |
| 28 | cytoplasmic vesicle (GO:0031410) | CC | 3.97 | down |
| 29 | positive regulation of cell differentiation (GO:0045597) | BP | 3.94 | down |
| 30 | cellular response to hypoxia (GO:0071456) | BP | 3.92 | up |
| 31 | negative regulation of ubiquitin protein ligase activity (GO:1904667) | BP | 3.90 | up |
| 32 | negative regulation of macroautophagy (GO:0016242) | BP | 3.85 | down |
| 33 | protein phosphorylation (GO:0006468) | BP | 3.83 | down |
| 34 | cellular glucose homeostasis (GO:0001678) | BP | 3.83 | down |
| 35 | mitochondrial respiratory chain complex assembly (GO:0033108) | BP | 3.80 | up |
| 36 | mitochondrial respiratory chain complex I biogenesis (GO:0097031) | BP | 3.73 | up |
| 37 | NADH dehydrogenase complex assembly (GO:0010257) | BP | 3.73 | up |
| 38 | mitochondrial respiratory chain complex I assembly (GO:0032981) | BP | 3.73 | up |
| 39 | regulation of cellular component organization (GO:0051128) | BP | 3.68 | down |
| 40 | regulation of fatty acid oxidation (GO:0046320) | BP | 3.64 | down |
| 41 | adenyl ribonucleotide binding (GO:0032559) | MF | 3.61 | down |
| 42 | cytoskeleton (GO:0005856) | CC | 3.52 | down |
| 43 | tropomyosin binding (GO:0005523) | MF | 3.50 | up |
| 44 | actin monomer binding (GO:0003785) | MF | 3.47 | up |
| 45 | negative regulation of ubiquitin-protein ligase activity involved in mitotic cell cycle (GO:0051436) | BP | 3.41 | up |
| 46 | positive regulation of wound healing (GO:0090303) | BP | 3.39 | down |
| 47 | response to ketone (GO:1901654) | BP | 3.39 | down |
| 48 | 3',5'-cyclic-AMP phosphodiesterase activity (GO:0004115) | MF | 3.37 | down |
| 49 | regulation of ubiquitin-protein ligase activity involved in mitotic cell cycle (GO:0051439) | BP | 3.37 | up |
| 50 | regulation of fat cell differentiation (GO:0045598) | BP | 3.37 | down |

**Table S7.** Top 50 significantly regulated biological pathways in response to SirT1 OE in young mice.

| **No** | **Pathway** | **GO term** | **log10P** | **Regulation** |
| --- | --- | --- | --- | --- |
| *SirT1 overexpression in young mice* | | | | |
| 1 | actin monomer binding (GO:0003785) | MF | 5.72 | up |
| 2 | extracellular matrix organization (GO:0030198) | BP | 5.27 | up |
| 3 | positive regulation of transcription, DNA-templated (GO:0045893) | BP | 4.89 | up |
| 4 | negative regulation of intracellular signal transduction (GO:1902532) | BP | 4.88 | up |
| 5 | protein kinase binding (GO:0019901) | MF | 4.66 | up |
| 6 | regulation of anatomical structure morphogenesis (GO:0022603) | BP | 4.43 | up |
| 7 | cellular response to nutrient levels (GO:0031669) | BP | 4.26 | down |
| 8 | regulation of I-kappaB kinase/NF-kappaB signaling (GO:0043122) | BP | 4.24 | up |
| 9 | protein phosphorylation (GO:0006468) | BP | 4.23 | down |
| 10 | collagen fibril organization (GO:0030199) | BP | 4.16 | up |
| 11 | regulation of transcription from RNA polymerase II promoter (GO:0006357) | BP | 4.12 | up |
| 12 | positive regulation of intracellular signal transduction (GO:1902533) | BP | 4.09 | up |
| 13 | protein kinase activity (GO:0004672) | MF | 3.99 | down |
| 14 | ubiquitin protein ligase binding (GO:0031625) | MF | 3.98 | down |
| 15 | platelet-derived growth factor binding (GO:0048407) | MF | 3.96 | up |
| 16 | cytoskeleton (GO:0005856) | CC | 3.89 | up |
| 17 | cellular glucose homeostasis (GO:0001678) | BP | 3.83 | down |
| 18 | regulation of cell migration (GO:0030334) | BP | 3.83 | up |
| 19 | lipid droplet (GO:0005811) | CC | 3.73 | down |
| 20 | ubiquitin-like protein ligase binding (GO:0044389) | MF | 3.73 | down |
| 21 | lipid homeostasis (GO:0055088) | BP | 3.69 | down |
| 22 | regulation of small GTPase mediated signal transduction (GO:0051056) | BP | 3.68 | up |
| 23 | transmembrane receptor protein serine/threonine kinase signaling pathway (GO:0007178) | BP | 3.56 | up |
| 24 | phosphatidylinositol phosphate phosphatase activity (GO:0052866) | MF | 3.53 | down |
| 25 | lipid particle organization (GO:0034389) | BP | 3.50 | down |
| 26 | chemical homeostasis (GO:0048878) | BP | 3.48 | down |
| 27 | cellular response to starvation (GO:0009267) | BP | 3.45 | down |
| 28 | kinase binding (GO:0019900) | MF | 3.42 | up |
| 29 | response to insulin (GO:0032868) | BP | 3.41 | down |
| 30 | positive regulation of transcription from RNA polymerase II promoter (GO:0045944) | BP | 3.40 | up |
| 31 | positive regulation of transcription from RNA polymerase II promoter (GO:0045944) | BP | 3.40 | down |
| 32 | negative regulation of protein kinase activity (GO:0006469) | BP | 3.39 | up |
| 33 | protein serine/threonine kinase activity (GO:0004674) | MF | 3.38 | down |
| 34 | cellular response to peptide hormone stimulus (GO:0071375) | BP | 3.36 | down |
| 35 | cellular response to insulin stimulus (GO:0032869) | BP | 3.36 | down |
| 36 | actin cytoskeleton (GO:0015629) | CC | 3.33 | up |
| 37 | glucose homeostasis (GO:0042593) | BP | 3.29 | down |
| 38 | negative regulation of lipid storage (GO:0010888) | BP | 3.26 | down |
| 39 | integral component of plasma membrane (GO:0005887) | CC | 3.25 | up |
| 40 | positive regulation of sprouting angiogenesis (GO:1903672) | BP | 3.23 | up |
| 41 | regulation of autophagy (GO:0010506) | BP | 3.21 | down |
| 42 | cell junction assembly (GO:0034329) | BP | 3.19 | up |
| 43 | regulation of extrinsic apoptotic signaling pathway (GO:2001236) | BP | 3.19 | up |
| 44 | muscle contraction (GO:0006936) | BP | 3.18 | down |
| 45 | focal adhesion (GO:0005925) | CC | 3.16 | up |
| 46 | positive regulation of cholesterol transport (GO:0032376) | BP | 3.15 | down |
| 47 | regulation of transcription from RNA polymerase II promoter (GO:0006357) | BP | 3.14 | down |
| 48 | regulation of apoptotic process (GO:0042981) | BP | 3.14 | up |
| 49 | protein kinase binding (GO:0019901) | MF | 3.12 | down |
| 50 | phosphatidylinositol-4,5-bisphosphate phosphatase activity (GO:0106019) | MF | 3.10 | down |

**Table S8.** Top 50 significantly regulated biological pathways in response to CR in young WT mice.

| **No** | **Pathway** | **GO term** | **log10P** | **Regulation** |
| --- | --- | --- | --- | --- |
| *WT20CR* | | | | |
| 1 | protein targeting to ER (GO:0045047) | BP | 19.20 | down |
| 2 | SRP-dependent cotranslational protein targeting to membrane (GO:0006614) | BP | 9.03 | down |
| 3 | cotranslational protein targeting to membrane (GO:0006613) | BP | 18.52 | down |
| 4 | cytosolic ribosome (GO:0022626) | CC | 18.45 | down |
| 5 | viral gene expression (GO:0019080) | BP | 17.70 | down |
| 6 | nuclear-transcribed mRNA catabolic process, nonsense-mediated decay (GO:0000184) | BP | 17.49 | down |
| 7 | viral transcription (GO:0019083) | BP | 17.39 | down |
| 8 | cytosolic part (GO:0044445) | CC | 16.40 | down |
| 9 | peptide biosynthetic process (GO:0043043) | BP | 15.31 | down |
| 10 | translation (GO:0006412) | BP | 14.40 | down |
| 11 | nuclear-transcribed mRNA catabolic process (GO:0000956) | BP | 13.50 | down |
| 12 | rRNA processing (GO:0006364) | BP | 12.72 | down |
| 13 | viral process (GO:0016032) | BP | 12.58 | down |
| 14 | cytosolic small ribosomal subunit (GO:0022627) | CC | 12.26 | down |
| 15 | small ribosomal subunit (GO:0015935) | CC | 11.70 | down |
| 16 | rRNA metabolic process (GO:0016072) | BP | 10.41 | down |
| 17 | ribosome (GO:0005840) | BP | 10.30 | down |
| 18 | cellular macromolecule biosynthetic process (GO:0034645) | BP | 9.62 | down |
| 19 | cellular protein metabolic process (GO:0044267) | BP | 9.28 | down |
| 20 | ribosome biogenesis (GO:0042254) | BP | 9.27 | down |
| 21 | cytoplasmic translation (GO:0002181) | BP | 9.26 | down |
| 22 | ncRNA processing (GO:0034470) | BP | 9.23 | down |
| 23 | muscle contraction (GO:0006936) | BP | 8.01 | down |
| 24 | cytosolic large ribosomal subunit (GO:0022625) | CC | 7.86 | down |
| 25 | gene expression (GO:0010467) | BP | 7.81 | down |
| 26 | actin-myosin filament sliding (GO:0033275) | BP | 7.66 | down |
| 27 | muscle filament sliding (GO:0030049) | BP | 7.66 | down |
| 28 | large ribosomal subunit (GO:0015934) | CC | 7.63 | down |
| 29 | mitochondrion (GO:0005739) | CC | 7.08 | down |
| 30 | mitochondrial matrix (GO:0005759) | CC | 6.57 | down |
| 31 | striated muscle contraction (GO:0006941) | BP | 6.54 | down |
| 32 | heart contraction (GO:0060047) | BP | 5.94 | down |
| 33 | focal adhesion (GO:0005925) | CC | 5.93 | down |
| 34 | regulation of muscle contraction (GO:0006937) | BP | 5.59 | down |
| 35 | transcription corepressor activity (GO:0003714) | MF | 5.43 | up |
| 36 | polysomal ribosome (GO:0042788) | CC | 5.36 | down |
| 37 | phosphatidylinositol binding (GO:0035091) | MF | 5.13 | up |
| 38 | ribosome assembly (GO:0042255) | BP | 5.02 | down |
| 39 | ribosomal small subunit biogenesis (GO:0042274) | BP | 4.94 | down |
| 40 | cellular glucose homeostasis (GO:0001678) | BP | 4.85 | down |
| 41 | glucose homeostasis (GO:0042593) | BP | 4.83 | down |
| 42 | 1-phosphatidylinositol binding (GO:0005545) | MF | 4.80 | up |
| 43 | cellular amino acid catabolic process (GO:0009063) | BP | 4.78 | down |
| 44 | myofibril assembly (GO:0030239) | BP | 4.72 | down |
| 45 | respiratory electron transport chain (GO:0022904) | BP | 4.64 | down |
| 46 | lipoprotein particle receptor activity (GO:0030228) | MF | 4.63 | up |
| 47 | acyl-CoA dehydrogenase activity (GO:0003995) | MF | 4.63 | down |
| 48 | regulation of cytoskeleton organization (GO:0051493) | BP | 4.60 | up |
| 49 | polysome (GO:0005844) | CC | 4.60 | down |
| 50 | cardiac muscle contraction (GO:0060048) | BP | 4.58 | down |

**Table S9.** Top 50 significantly regulated biological pathways in response to CR in young SirT1 KO mice.

| **No** | **Pathway** | **GO term** | **log10P** | **Regulation** |
| --- | --- | --- | --- | --- |
| *KO20CR* | | | | |
| 1 | mitochondrial ATP synthesis coupled electron transport (GO:0042775) | BP | 11.56 | down |
| 2 | muscle contraction (GO:0006936) | BP | 11.42 | up |
| 3 | actin cytoskeleton (GO:0015629) | CC | 10.81 | up |
| 4 | respiratory electron transport chain (GO:0022904) | BP | 10.78 | down |
| 5 | alpha-actinin binding (GO:0051393) | MF | 8.94 | up |
| 6 | actin binding (GO:0003779) | MF | 8.88 | up |
| 7 | regulation of fat cell differentiation (GO:0045598) | BP | 8.61 | up |
| 8 | actomyosin structure organization (GO:0031032) | BP | 7.70 | up |
| 9 | mitochondrial inner membrane (GO:0005743) | CC | 7.39 | down |
| 10 | NADH dehydrogenase (quinone) activity (GO:0050136) | MF | 7.10 | down |
| 11 | NADH dehydrogenase (ubiquinone) activity (GO:0008137) | MF | 7.10 | down |
| 12 | cytoskeleton (GO:0005856) | CC | 6.91 | up |
| 13 | mitochondrial electron transport, NADH to ubiquinone (GO:0006120) | BP | 6.80 | down |
| 14 | positive regulation of fat cell differentiation (GO:0045600) | BP | 6.41 | up |
| 15 | mitochondrial respiratory chain complex I (GO:0005747) | CC | 6.35 | down |
| 16 | NADH dehydrogenase complex assembly (GO:0010257) | BP | 6.32 | down |
| 17 | mitochondrial respiratory chain complex I biogenesis (GO:0097031) | BP | 6.32 | down |
| 18 | mitochondrial respiratory chain complex I assembly (GO:0032981) | BP | 6.32 | down |
| 19 | sarcomere organization (GO:0045214) | BP | 6.16 | up |
| 20 | myofibril assembly (GO:0030239) | BP | 5.68 | up |
| 21 | cardiac muscle tissue morphogenesis (GO:0055008) | BP | 5.63 | up |
| 22 | actomyosin (GO:0042641) | CC | 5.60 | up |
| 23 | actin filament binding (GO:0051015) | MF | 5.44 | up |
| 24 | muscle filament sliding (GO:0030049) | BP | 5.44 | up |
| 25 | actin-myosin filament sliding (GO:0033275) | BP | 5.44 | up |
| 26 | muscle tissue morphogenesis (GO:0060415) | BP | 5.31 | up |
| 27 | mitochondrial respiratory chain complex assembly (GO:0033108) | BP | 5.27 | down |
| 28 | negative regulation of cell differentiation (GO:0045596) | BP | 5.25 | up |
| 29 | cardiac muscle fiber development (GO:0048739) | BP | 5.18 | up |
| 30 | connective tissue development (GO:0061448) | BP | 5.14 | up |
| 31 | negative regulation of angiogenesis (GO:0016525) | BP | 5.10 | up |
| 32 | positive regulation of cell differentiation (GO:0045597) | BP | 5.09 | up |
| 33 | sarcoplasm (GO:0016528) | CC | 5.04 | up |
| 34 | mitochondrial electron transport, cytochrome c to oxygen (GO:0006123) | BP | 5.03 | down |
| 35 | regulation of angiogenesis (GO:0045765) | BP | 5.00 | up |
| 36 | response to insulin (GO:0032868) | BP | 4.99 | up |
| 37 | negative regulation of macrophage derived foam cell differentiation (GO:0010745) | BP | 4.98 | up |
| 38 | striated muscle myosin thick filament assembly (GO:0071688) | BP | 4.98 | up |
| 39 | negative regulation of cell migration (GO:0030336) | BP | 4.97 | up |
| 40 | actinin binding (GO:0042805) | MF | 4.90 | up |
| 41 | skeletal muscle myosin thick filament assembly (GO:0030241) | BP | 4.90 | up |
| 42 | negative regulation of cell adhesion mediated by integrin (GO:0033629) | BP | 4.90 | up |
| 43 | negative regulation of intracellular signal transduction (GO:1902532) | BP | 4.88 | up |
| 44 | titin binding (GO:0031432) | MF | 4.80 | up |
| 45 | negative regulation of endothelial cell migration (GO:0010596) | BP | 4.76 | up |
| 46 | striated muscle contraction (GO:0006941) | BP | 4.71 | up |
| 47 | regulation of sodium ion transport (GO:0002028) | BP | 4.67 | up |
| 48 | regulation of transcription from RNA polymerase II promoter (GO:0006357) | BP | 4.66 | up |
| 49 | cardiac muscle tissue development (GO:0048738) | BP | 4.58 | up |
| 50 | cytoplasmic vesicle (GO:0031410) | CC | 4.51 | up |

**Table S10.** Top 50 significantly regulated biological pathways following CR in young SirT1 OE mice.

| **No** | **Pathway** | **GO term** | **log10P** | **Regulation** |
| --- | --- | --- | --- | --- |
| *OE20CR* | | | | |
| 1 | extracellular matrix organization (GO:0030198) | BP | 22.69 | down |
| 2 | RNA binding (GO:0003723) | MF | 12.68 | up |
| 3 | collagen fibril organization (GO:0030199) | BP | 11.73 | down |
| 4 | collagen binding (GO:0005518) | MF | 10.64 | down |
| 5 | protein complex subunit organization (GO:0071822) | BP | 10.35 | down |
| 6 | regulation of cell migration (GO:0030334) | BP | 10.01 | down |
| 7 | nuclear-transcribed mRNA catabolic process, nonsense-mediated decay (GO:0000184) | BP | 9.46 | up |
| 8 | focal adhesion (GO:0005925) | CC | 9.34 | down |
| 9 | SRP-dependent cotranslational protein targeting to membrane (GO:0006614) | BP | 9.22 | up |
| 10 | translation factor activity, RNA binding (GO:0008135) | MF | 9.04 | up |
| 11 | endoplasmic reticulum lumen (GO:0005788) | CC | 8.97 | down |
| 12 | cotranslational protein targeting to membrane (GO:0006613) | BP | 8.92 | up |
| 13 | platelet-derived growth factor binding (GO:0048407) | MF | 8.75 | down |
| 14 | protein targeting to ER (GO:0045047) | BP | 8.65 | up |
| 15 | translation initiation factor activity (GO:0003743) | MF | 7.91 | up |
| 16 | viral gene expression (GO:0019080) | BP | 7.83 | up |
| 17 | nuclear-transcribed mRNA catabolic process (GO:0000956) | BP | 7.82 | up |
| 18 | viral transcription (GO:0019083) | BP | 7.66 | up |
| 19 | vacuolar lumen (GO:0005775) | CC | 7.65 | down |
| 20 | lysosomal lumen (GO:0043202) | CC | 7.57 | down |
| 21 | regulation of translational initiation (GO:0006446) | BP | 7.44 | up |
| 22 | viral process (GO:0016032) | BP | 7.42 | up |
| 23 | ribosome biogenesis (GO:0042254) | BP | 7.21 | up |
| 24 | cytosolic ribosome (GO:0022626) | CC | 7.08 | up |
| 25 | positive regulation of cell migration (GO:0030335) | BP | 6.74 | down |
| 26 | rRNA processing (GO:0006364) | BP | 6.73 | up |
| 27 | rRNA processing (GO:0006364) | CC | 6.69 | up |
| 28 | peptide biosynthetic process (GO:0043043) | BP | 6.40 | up |
| 29 | translation (GO:0006412) | BP | 6.38 | up |
| 30 | regulation of translation (GO:0006417) | BP | 6.36 | up |
| 31 | positive regulation of cell motility (GO:2000147) | BP | 6.22 | down |
| 32 | rRNA metabolic process (GO:0016072) | BP | 6.15 | up |
| 33 | cytosolic large ribosomal subunit (GO:0022625) | CC | 5.98 | up |
| 34 | ncRNA processing (GO:0034470) | BP | 5.92 | up |
| 35 | actin cytoskeleton (GO:0015629) | CC | 5.81 | down |
| 36 | cytosolic part (GO:0044445) | CC | 5.60 | up |
| 37 | ribosome (GO:0005840) | CC | 5.55 | up |
| 38 | extracellular matrix disassembly (GO:0022617) | BP | 5.44 | down |
| 39 | positive regulation of fibroblast proliferation (GO:0048146) | BP | 5.36 | down |
| 40 | gene expression (GO:0010467) | BP | 5.26 | up |
| 41 | keratan sulfate catabolic process (GO:0042340) | BP | 5.18 | down |
| 42 | cytoskeleton (GO:0005856) | CC | 5.09 | down |
| 43 | mitochondrial ATP synthesis coupled electron transport (GO:0042775) | BP | 5.07 | down |
| 44 | lysosome (GO:0005764) | CC | 5.05 | down |
| 45 | regulation of phosphorylation (GO:0042325) | BP | 5.05 | down |
| 46 | membrane raft (GO:0045121) | CC | 5.05 | down |
| 47 | ribonucleoprotein complex assembly (GO:0022618) | BP | 5.05 | up |
| 48 | mitochondrial ATP synthesis coupled proton transport (GO:0042776) | BP | 5.03 | down |
| 49 | endodermal cell differentiation (GO:0035987) | BP | 4.94 | down |
| 50 | positive regulation of phosphorylation (GO:0042327) | BP | 4.70 | down |

**Table S11.** Top 50 significantly regulated biological pathways in response to CR in old WT mice.

| **No** | **Pathway** | **GO term** | **log10P** | **Regulation** |
| --- | --- | --- | --- | --- |
| *WT80CR* | | | | |
| 1 | SRP-dependent cotranslational protein targeting to membrane (GO:0006614) | BP | 15.51 | up |
| 2 | cotranslational protein targeting to membrane (GO:0006613) | BP | 15.08 | up |
| 3 | protein targeting to ER (GO:0045047) | BP | 14.66 | up |
| 4 | viral transcription (GO:0019083) | BP | 13.19 | up |
| 5 | rRNA processing (GO:0006364) | BP | 12.72 | up |
| 6 | viral gene expression (GO:0019080) | BP | 12.44 | up |
| 7 | cytosolic ribosome (GO:0022626) | CC | 12.33 | up |
| 8 | nuclear-transcribed mRNA catabolic process, nonsense-mediated decay (GO:0000184) | BP | 12.28 | up |
| 9 | ribosome biogenesis (GO:0042254) | BP | 11.50 | up |
| 10 | rRNA metabolic process (GO:0016072) | BP | 11.20 | up |
| 11 | cytosolic part (GO:0044445) | CC | 10.94 | up |
| 12 | ncRNA processing (GO:0034470) | BP | 10.69 | up |
| 13 | nuclear-transcribed mRNA catabolic process (GO:0000956) | BP | 10.12 | up |
| 14 | RNA binding (GO:0003723) | MF | 9.36 | up |
| 15 | peptide biosynthetic process (GO:0043043) | BP | 9.33 | up |
| 16 | large ribosomal subunit (GO:0015934) | CC | 8.61 | up |
| 17 | cytoplasmic translation (GO:0002181) | BP | 8.16 | up |
| 18 | cytosolic large ribosomal subunit (GO:0022625) | CC | 7.86 | up |
| 19 | gene expression (GO:0010467) | BP | 7.27 | up |
| 20 | extracellular matrix organization (GO:0030198) | BP | 7.11 | down |
| 21 | translation (GO:0006412) | BP | 7.01 | up |
| 22 | viral process (GO:0016032) | BP | 6.77 | up |
| 23 | ribosome (GO:0005840) | CC | 5.55 | up |
| 24 | focal adhesion (GO:0005925) | CC | 5.42 | down |
| 25 | endoplasmic reticulum lumen (GO:0005788) | CC | 5.32 | down |
| 26 | transcription corepressor activity (GO:0003714) | MF | 4.83 | down |
| 27 | nucleolus (GO:0005730) | CC | 4.74 | up |
| 28 | cellular macromolecule biosynthetic process (GO:0034645) | BP | 4.71 | up |
| 29 | interleukin-1-mediated signaling pathway (GO:0070498) | BP | 4.60 | up |
| 30 | cytosolic small ribosomal subunit (GO:0022627) | CC | 4.58 | up |
| 31 | transcription regulatory region DNA binding (GO:0044212) | MF | 4.58 | down |
| 32 | membrane raft (GO:0045121) | CC | 4.36 | down |
| 33 | small ribosomal subunit (GO:0015935) | CC | 4.32 | up |
| 34 | regulation of RNA splicing (GO:0043484) | BP | 4.20 | up |
| 35 | collagen fibril organization (GO:0030199) | BP | 4.16 | down |
| 36 | RNA splicing (GO:0008380) | BP | 4.16 | up |
| 37 | RNA splicing, via transesterification reactions with bulged adenosine as nucleophile (GO:0000377) | BP | 4.01 | up |
| 38 | platelet-derived growth factor binding (GO:0048407) | MF | 3.96 |  |
| 39 | integrin binding (GO:0005178) | MF | 3.92 | down |
| 40 | negative regulation of ubiquitin protein ligase activity (GO:1904667) | BP | 3.90 | up |
| 41 | cytoskeleton (GO:0005856) | CC | 3.89 | down |
| 42 | regulation of cellular ketone metabolic process (GO:0010565) | BP | 3.88 | up |
| 43 | regulation of cell migration (GO:0030334) | BP | 3.83 | down |
| 44 | positive regulation of cell migration (GO:0030335) | BP | 3.83 | down |
| 45 | actin cytoskeleton (GO:0015629) | CC | 3.78 | down |
| 46 | cellular protein metabolic process (GO:0044267) | BP | 3.63 | up |
| 47 | negative regulation of cytoskeleton organization (GO:0051494) | BP | 3.56 | down |
| 48 | mRNA processing (GO:0006397) | BP | 3.53 | up |
| 49 | mRNA splicing, via spliceosome (GO:0000398) | BP | 3.49 | up |
| 50 | actin filament binding (GO:0051015) | MF | 3.46 | down |

**Table S12.** Top 50 significantly regulated biological pathways in response to CR in old SirT1 KO mice.

| **No** | **Pathway** | **GO term** | **log10P** | **Regulation** |
| --- | --- | --- | --- | --- |
| *KO80CR* | | | | |
| 1 | regulation of transcription from RNA polymerase II promoter (GO:0006357) | BP | 10.03 | down |
| 2 | mitochondrial matrix (GO:0005759) | CC | 8.98 | up |
| 3 | mitochondrion (GO:0005739) | CC | 8.68 | up |
| 4 | fatty acid beta-oxidation (GO:0006635) | BP | 8.57 | up |
| 5 | RNA polymerase II regulatory region sequence-specific DNA binding (GO:0000977) | MF | 7.70 | down |
| 6 | negative regulation of transcription from RNA polymerase II promoter (GO:0000122) | BP | 7.44 | down |
| 7 | fatty acid catabolic process (GO:0009062) | BP | 7.20 | up |
| 8 | cytosolic ribosome (GO:0022626) | CC | 7.08 | down |
| 9 | long-chain fatty acid transport (GO:0015909) | BP | 6.97 | up |
| 10 | positive regulation of transcription, DNA-templated (GO:0045893) | BP | 6.93 | down |
| 11 | muscle filament sliding (GO:0030049) | BP | 6.52 | up |
| 12 | actin-myosin filament sliding (GO:0033275) | BP | 6.52 | up |
| 13 | SRP-dependent cotranslational protein targeting to membrane (GO:0006614) | BP | 6.50 | down |
| 14 | RNA binding (GO:0003723) | MF | 6.48 | down |
| 15 | regulation of fatty acid oxidation (GO:0046320) | BP | 6.46 | up |
| 16 | nuclear-transcribed mRNA catabolic process, nonsense-mediated decay (GO:0000184) | BP | 6.10 | down |
| 17 | rRNA processing (GO:0006364) | BP | 6.08 | down |
| 18 | heart contraction (GO:0060047) | BP | 5.94 | up |
| 19 | negative regulation of transcription, DNA-templated (GO:0045892) | BP | 5.86 | down |
| 20 | cytosolic part (GO:0044445) | CC | 5.60 | down |
| 21 | rRNA metabolic process (GO:0016072) | BP | 5.52 | down |
| 22 | RNA polymerase II regulatory region DNA binding (GO:0001012) | MF | 5.49 | down |
| 23 | cotranslational protein targeting to membrane (GO:0006613) | BP | 5.46 | down |
| 24 | fatty acid oxidation (GO:0019395) | BP | 5.45 | up |
| 25 | viral gene expression (GO:0019080) | BP | 5.43 | down |
| 26 | transcription corepressor activity (GO:0003714) | MF | 5.43 | down |
| 27 | ribosome biogenesis (GO:0042254) | BP | 5.36 | down |
| 28 | ncRNA processing (GO:0034470) | BP | 5.33 | down |
| 29 | viral transcription (GO:0019083) | BP | 5.30 | down |
| 30 | protein targeting to ER (GO:0045047) | BP | 5.27 | down |
| 31 | cytoplasmic translation (GO:0002181) | BP | 5.16 | down |
| 32 | cytosolic large ribosomal subunit (GO:0022625) | CC | 5.10 | down |
| 33 | nuclear-transcribed mRNA catabolic process (GO:0000956) | BP | 5.09 | down |
| 34 | muscle contraction (GO:0006936) | BP | 5.05 | up |
| 35 | transcription regulatory region DNA binding (GO:0044212) | MF | 5.04 | down |
| 36 | RNA polymerase II core promoter proximal region sequence-specific DNA binding (GO:0000978) | MF | 4.98 | down |
| 37 | large ribosomal subunit (GO:0015934) | CC | 4.93 | down |
| 38 | transcription regulatory region sequence-specific DNA binding (GO:0000976) | MF | 4.80 | down |
| 39 | E-box binding (GO:0070888) | MF | 4.76 | down |
| 40 | ribosome (GO:0005840) | CC | 4.72 | down |
| 41 | striated muscle contraction (GO:0006941) | BP | 4.71 | up |
| 42 | regulation of transcription from RNA polymerase II promoter (GO:0045944) | BP | 4.68 | down |
| 43 | fatty acid beta-oxidation using acyl-CoA dehydrogenase (GO:0033539) | BP | 4.63 | up |
| 44 | core promoter proximal region sequence-specific DNA binding (GO:0000987) | MF | 4.60 | down |
| 45 | cardiac muscle contraction (GO:0060048) | BP | 4.58 | up |
| 46 | core promoter binding (GO:0001047) | MF | 4.51 | down |
| 47 | regulation of cardiac muscle cell contraction (GO:0086004) | BP | 4.45 | up |
| 48 | contractile fiber (GO:0043292) | CC | 4.45 | up |
| 49 | regulation of cardiac conduction (GO:1903779) | BP | 4.38 | up |
| 50 | positive regulation of triglyceride biosynthetic process (GO:0010867) | BP | 4.36 | down |

**Table S13.** Top 50 significantly regulated biological pathways in response to CR in old SirT1 OE mice.

| **No** | **Pathway** | **GO term** | **log10P** | **Regulation** |
| --- | --- | --- | --- | --- |
| *OE80CR* | | | | |
| 1 | focal adhesion (GO:0005925) | CC | 12.61 | down |
| 2 | muscle filament sliding (GO:0030049) | BP | 11.41 | down |
| 3 | actin-myosin filament sliding (GO:0033275) | BP | 11.41 | down |
| 4 | extracellular matrix organization (GO:0030198) | BP | 11.36 | down |
| 5 | endoplasmic reticulum lumen (GO:0005788) | CC | 8.32 | down |
| 6 | integrin binding (GO:0005178) | MF | 7.94 | down |
| 7 | collagen fibril organization (GO:0030199) | BP | 7.64 | down |
| 8 | muscle contraction (GO:0006936) | BP | 7.23 | down |
| 9 | platelet-derived growth factor binding (GO:0048407) | MF | 7.01 | down |
| 10 | protein complex subunit organization (GO:0071822) | BP | 6.90 | down |
| 11 | actomyosin structure organization (GO:0031032) | BP | 6.76 | down |
| 12 | protein ubiquitination (GO:0016567) | BP | 6.71 | up |
| 13 | myofibril assembly (GO:0030239) | BP | 6.71 | down |
| 14 | actomyosin (GO:0042641) | CC | 6.61 | down |
| 15 | ubiquitin-protein transferase activity (GO:0004842) | MF | 6.61 | up |
| 16 | actin cytoskeleton (GO:0015629) | CC | 6.37 | down |
| 17 | protein polyubiquitination (GO:0000209) | BP | 6.10 | up |
| 18 | post-translational protein modification (GO:0043687) | BP | 5.90 | up |
| 19 | lysosomal transport (GO:0007041) | BP | 5.79 | up |
| 20 | ubiquitin-dependent protein catabolic process (GO:0006511) | BP | 5.75 | up |
| 21 | actin binding (GO:0003779) | MF | 5.74 | down |
| 22 | actin monomer binding (GO:0003785) | MF | 5.72 | down |
| 23 | modification-dependent protein catabolic process (GO:0019941) | BP | 5.59 | up |
| 24 | collagen binding (GO:0005518) | MF | 5.30 | down |
| 25 | stress fiber (GO:0001725) | CC | 5.26 | down |
| 26 | contractile actin filament bundle (GO:0097517) | CC | 5.26 | down |
| 27 | regulation of blood coagulation (GO:0030193) | BP | 5.14 | down |
| 28 | regulation of sequestered release of calcium ion into cytosol (GO:0051281) | BP | 5.04 | down |
| 29 | lysosomal lumen (GO:0043202) | CC | 5.02 | down |
| 30 | regulation of cellular component organization (GO:0051128) | BP | 4.94 | up |
| 31 | heart contraction (GO:0060047) | BP | 4.94 | down |
| 32 | actin filament binding (GO:0051015) | MF | 4.74 | down |
| 33 | endosome to lysosome transport (GO:0008333) | BP | 4.65 | up |
| 34 | response to leptin (GO:0044321) | BP | 4.60 | down |
| 35 | endosome organization (GO:0007032) | BP | 4.58 | up |
| 36 | regulation of endothelial cell proliferation (GO:0001936) | BP | 4.57 | down |
| 37 | ubiquitin protein ligase activity (GO:0061630) | MF | 4.56 | up |
| 38 | calcium ion binding (GO:0005509) | MF | 4.47 | down |
| 39 | regulation of muscle contraction (GO:0006937) | BP | 4.45 | down |
| 40 | contractile fiber (GO:0043292) | CC | 4.45 | down |
| 41 | positive regulation of cell migration (GO:0030335) | BP | 4.36 | down |
| 42 | myofibril (GO:0030016) | CC | 4.35 | down |
| 43 | cellular protein modification process (GO:0006464) | BP | 4.27 | up |
| 44 | actin-based cell projection (GO:0098858) | CC | 4.22 | down |
| 45 | lysine-acetylated histone binding (GO:0070577) | MF | 4.20 | up |
| 46 | regulation of smooth muscle cell migration (GO:0014910) | BP | 4.20 | down |
| 47 | microfilament motor activity (GO:0000146) | MF | 4.20 | down |
| 48 | acetylation-dependent protein binding (GO:0140033) | MF | 4.20 | up |
| 49 | filopodium tip (GO:0032433) | CC | 4.14 | up |
| 50 | skeletal muscle thin filament assembly (GO:0030240) | BP | 4.14 | down |
